# Hyperthyroidism unmasked several years after the medical and radiosurgical treatment of an invasive macroprolactinoma inducing hypopituitarism: a case report

**DOI:** 10.4076/1757-1626-2-6449

**Published:** 2009-07-29

**Authors:** Luca Foppiani, Antonio Ruelle, Paolo Cavazzani, Patrizia del Monte

**Affiliations:** 1 Department of Endocrinology, Galliera HospitalMura Delle Cappuccine 14, 16128 GenovaItaly; 2 Unit of Neurosurgery, Galliera HospitalMura Delle Cappuccine 14, 16128 GenovaItaly; 3 Unit of Stereotactic Radiosurgery, Galliera HospitalMura Delle Cappuccine 14, 16128 GenovaItaly

## Abstract

**Introduction:**

Measuring thyroid stimulating hormone levels alone may be insufficient to appropriately evaluate thyroid function. Reduced thyroid stimulating hormone levels associated to normal/reduced FT4 levels should prompt investigation of pituitary function, on suspicion of hypopituitarism. Pituitary macroadenomas are the most common cause of hypopituitarism; among these, macroprolactinomas are usually treated with dopamine-agonist therapy. Hypopituitarism does not preclude the development of primary hyperthyroidism. This report describes the case of a patient with a final diagnosis of macroprolactinoma inducing hypopituitarism, who subsequently developed hyperthyroidism due to a toxic thyroid nodule.

**Case presentation:**

A 62-year-old man underwent biochemistry and thyroid function assessment for asthenia. Reduced thyroid stimulating hormone levels were associated to slightly decreased FT4 levels and low-normal FT3 levels; thyroid ultrasonography showed a multinodular goiter. Thyroid scan with ^99m^Tc-pertechnetate revealed an autonomous left nodule with suppression of the surrounding parenchyma. Pituitary investigation showed partial hypopituitarism associated to increased prolactin levels: 182-200 ng/ml. Magnetic resonance imaging revealed a large (2.2 cm) invasive macroadenoma. To avoid a possible high-dose hook effect, the patient's serum was diluted; the resulting PRL levels of around 1800 ng/ml prompted the final diagnosis of macroprolactinoma. Reduced libido and erectile dysfunction were ascertained. In addition to replacement therapy with L-thyroxine and testosterone, cabergoline was started and was progressively increased to high doses (4 g/week); this yielded a significant but incomplete reduction of PRL levels (63-99 ng/ml). Sexual function improved. The macroadenoma shrank over the first two years of therapy, but subsequently enlarged slightly. Following stereotactic radiosurgery, the tumor stabilized and prolactin almost normalized (22 ng/ml) on therapy. Over the years, thyroid nodule volume was unmodified, but hyperthyroidism on L-thyroxine therapy was found, and increased FT3 levels with suppressed thyroid stimulating hormone levels were confirmed off-therapy. Thyroid scan confirmed the left autonomous nodule, which was successfully treated with methimazole.

**Conclusion:**

Reduced thyroid stimulating hormone levels associated to normal/reduced free-thyroid hormone levels may be the first clue to unsuspected hypopituitarism. Moderately increased prolactin levels in the presence of a large macroadenoma warrant serum dilution in order to avoid a possible hook effect. Stereotactic radiosurgery is a useful non-invasive tool in the management of pituitary tumors. A pre-toxic thyroid nodule with low secretory activity may initially be masked by the coexistence of secondary hypothyroidism, but may lead to overt hyperthyroidism over time.

## Introduction

Decreased/normal TSH levels associated to low-normal/reduced free-thyroid hormone levels may be the first sign of undiagnosed hypopituitarism (HYPO) [1-3]. HYPO is frequently due to pituitary macroadenomas. Among these, macroprolactinomas are usually managed by means of long-acting dopamine agonist therapy [4-6]. Nevertheless, around 20% of macroprolactinomas are resistant to medical therapy in terms of both lack of PRL normalization and tumor shrinkage [4-7]. A high-dose hook effect (the antibody used in the two-site-assays is saturated by very high PRL levels) should be considered whenever a huge pituitary adenoma is associated to modestly increased PRL levels [4]. Tailored stereotactic radiosurgery usually allows satisfactory non-invasive management of invasive pituitary adenomas resistant to medical therapy or not amenable or refractory to surgery, in terms of hormonal control and volume reduction [8-10]; nevertheless, the results in medically and surgically refractory prolactinomas are modest [9]. Here, we describe the case of a patient who was eventually diagnosed as having an invasive macroprolactinoma, which caused hypopituitarism and was partially resistant to cabergoline; the tumor was satisfactorily managed through a combination of medical treatment and radiosurgery. Over the years, the initial secondary hypothyroidism turned into primary hyperthyroidism due to a toxic thyroid nodule.

## Case presentation

**Figure 1.**
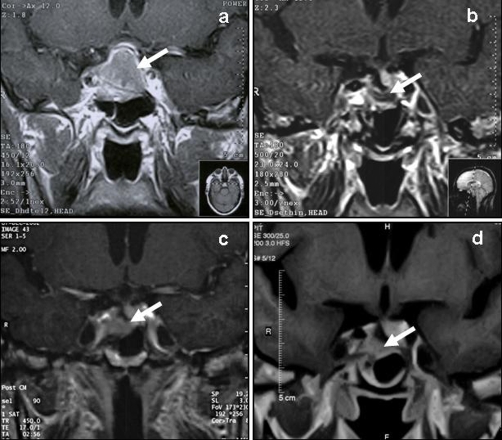
Magnetic resonance imaging scans of the pituitary macroadenoma over the years in the patient studied. Contrast-enhanced coronal T1-weighted magnetic resonance imaging showing a large pituitary macroadenoma (arrow) invading the right cavernous sinus **(a)**. After one year of cabergoline therapy the adenoma was markedly reduced and the intrasellar remnant (arrow) was modest **(b)**. After two years of therapy a minimal, but evident, increase in the intrasellar remnant (arrow) was observed **(c)**. Two years after stereotactic radiosurgery the intrasellar remnant (arrow) was stable **(d)**.

**Figure 2.**
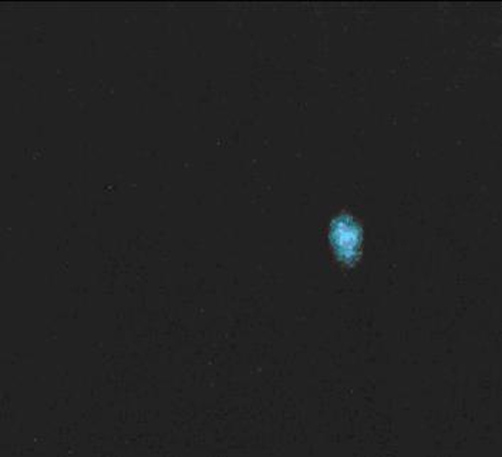
The patient's last ^99m^Tc-pertechnetate thyroid scan showing an autonomous left nodule with complete suppression of the surrounding parenchyma. Thyroid function assessment showed moderate hyperthyroidism.

In 1999, a 62-year-old hypertensive Caucasian man was referred to our out-patient endocrinology clinic following the finding of reduced TSH levels: 0.1 µU/ml (n.v. 0.27-4.2) associated to slightly decreased FT4 levels: 0.6 ng/dl (n.v. 0.7-1.7) during routine follow-up examinations for asthenia. The general practitioner had advised a ^99^Tc-pertechnetate thyroid scan, which showed a left autonomous nodule with inhibition of the surrounding parenchyma, and a thyroid ultrasonography (USG), which revealed a multinodular goiter (nodule diameter range: 15-25 mm). The patient's anamnesis was unremarkable apart from right cryptorchidism (only the left testis was palpable in the scrotum). Given the hormonal findings, a general basal assessment of pituitary function was performed. This revealed partial hypopituitarism (HYPO): hypogonadotropic hypogonadism (undetectable LH <0.5 IU/L and reduced FSH: 1.5 IU/L levels, undetectable testosterone levels: <0.2 ng/ml (n.v. 3-9)) and confirmed secondary hypothyroidism (reduced TSH levels: 0.2 µIU/ml associated to slightly decreased FT4 levels: 0.6 (n.v. 0.7-1.7) and low-normal FT3 levels), and undetectable GH with markedly reduced IGF-I levels: 15 ng/ml (n.v. 100-250 ng/ml for patient's age). Cortisol levels were normal: 14 µg/dl, whereas PRL levels were increased: 182-200 ng/ml (monomeric form) (n.v. 3-20). Given the clear diagnosis of partial hypopituitarism by means of baseline hormonal evaluation, dynamic pituitary assessments were not performed. Contrast-enhanced MRI (magnetic resonance imaging) examination of the brain showed a large pituitary macroadenoma (vertical diameter 2.2 cm), which invaded the right cavernous sinus, the sphenoidal sinus and the optic apparatus (Figrue 1a). Visual field examination was normal. Owing to the possible discrepancy between PRL levels and the volume of the tumor, serial dilution of the patient's serum was performed in order to avoid the hook effect, and a final PRL level of 1773 ng/ml was obtained. A diagnosis of HYPO secondary to a large macroprolactinoma was eventually made. A targeted anamnesis ascertained erectile dysfunction and a reduction in libido over the previous two years. In agreement with the neurosurgeon, medical treatment with cabergoline at the initial dose of 1 mg/week was begun. In addition, replacement therapy with L-thyroxine (75 µg/day) and parenteral depot testosterone (250 mg i.m./3 weeks) was started, which normalized FT4 and testosterone levels over time. Fine-needle aspiration biopsy of thyroid nodules disclosed a cytological pattern compatible with goiter. PRL levels two months after the start of therapy were significantly reduced: 377 ng/ml (Δ%: −78%) in comparison with the baseline, and MRI showed a slight shrinkage of the macroadenoma (not shown). Cabergoline was progressively increased over time, up to a maximal dosage of 4 mg/week; PRL levels declined further but did not normalize (range: 63-99 ng/ml). Libido and sexual capability improved. MRI documented a progressive significant reduction in the pituitary mass (intrasellar remnant) over the first two years (Figure 1b); thereafter, the intrasellar remnant enlarged slightly (Figure 1c) (PRL levels at that time were 99 ng/ml). Cortisol levels (14-18 µg/dl, normal) and IGF-I levels (<30 ng/ml, reduced) remained unchanged. The combination of (slight) tumor enlargement and lack of normalization of PRL levels on medical therapy prompted us to perform stereotactic radiosurgery (SRS) (total dose 2000 cGy). A few months later, thrombocytopenia occurred (35000-47000/mm^3^). Bone marrow evaluation by means of iliac bone biopsy showed normal hematopoiesis; antiplatelet autoantibodies were negative. Thrombocytopenia was managed conservatively with short-term cortisone therapy. The platelet count increased over time, but remained low (last value: 98000/mm^3^). Over the 5 years following SRS, PRL levels progressively declined, approaching normalization (last PRL levels: 22 ng/ml), on high-dose cabergoline (3 mg/week), and the tumor volume remained stable (Figure 1d). Since high-dosages of the dopamine-agonist drug were used, an echocardiogram was performed; this proved normal. Cortisol levels remained normal as well as FT4 and FT3 levels on L-thyroxine replacement therapy. Thyroid USG did not show significant variations in thyroid nodule volume over the years. At the end of 2007, thyroid hormone assay on L-thyroxine 75 µg/day surprisingly showed FT4 levels in the upper normal range: 1.7 ng/dl (n.v. 0.7-1.7), increased FT3 levels: 5.8 pg/ml (n.v. 1.8-4.6) and suppressed TSH levels: <0.01 µIU/ml. The patient also complained of palpitations. Although L-thyroxine was tapered to 50 µg/day, FT3 levels were unmodified two months later; the drug was therefore withdrawn and moderate FT3 (5.4 pg/ml) hyperthyroidism (with FT4 levels in the upper normal range: 1.5 ng/dl) was confirmed; thyroid auto-antibodies and anti-TSH-receptor antibodies were negative. A thyroid scan showed the initial picture (seen 8 years earlier) of a left autonomous nodule (Figure 2). As the patient refused surgery or radioiodine therapy, low-dose (5 mg/day) methimazole was started. This normalized FT3 levels, whereas TSH levels remained low. The patient is scheduled for regular hormonal and morphological follow-up.

## Discussion

HYPO is a rare condition and may be overlooked. The clinical symptoms may range from subtle and unspecific to dramatic, as when pituitary apoplexia occurs. Macroadenomas are the most prevalent cause of HYPO, though it is being increasingly acknowledged that brain damage of different etiologies (e.g. traumas, aneurysmal subarachnoid hemorrhage and ischemic stroke) is a significant cause of hypothalamic-pituitary dysfunction [1]. HYPO is mostly due to non-secreting macroadenomas; among secreting tumors, macroprolactinomas are the most common and are more frequent in men [4,5,7]. These tumors can cause mass-related symptoms and symptoms related to hypogonadism, which is usually diagnosed late in men [4,7]. In our patient, who was eventually diagnosed as having a huge macroprolactinoma causing HYPO, an impairment of sexual function was ascertained only by means of a targeted anamnesis. The final diagnosis was made after hormonal and morphological investigation following the finding of reduced TSH levels associated to slightly reduced FT4. Surprisingly, these findings were associated to an autonomous thyroid nodule, as revealed by thyroid scan. PRL levels were only moderately elevated and associated to a voluminous tumor mass. To avoid a possible high-dose hook effect [4], the patient's serum was appropriately diluted; this strategy revealed final real PRL levels about ten-fold higher (~1800ng/ml). This is a crucial point in patient management, since macroprolactinomas, unlike non-secreting macroadenomas, usually respond very well to dopaminergic therapy in terms of volume shrinkage and reduction of PRL levels, thus obviating the need for surgery in most cases. In our patient, secondary hypothyroidism and hypogonadism were successfully treated with replacement therapy, whereas the macroprolactinoma and related hyperprolactinemia were managed with the long-acting dopamine-agonist cabergoline. However, high-doses (4 mg/week) were required in order to obtain a significant, though incomplete, reduction in PRL levels. Resistance to dopaminergic therapy occurs in around 10-20% of prolactinomas and seems to be related to a loss of D2 receptors in tumor lactotrophs [4-7]. In our patient, after partial shrinkage over the first two years of therapy, the tumor enlarged. SRS was therefore performed, which achieved satisfactory control of the tumor over time (5-year follow-up) and led to almost complete normalization of PRL on cabergoline therapy. Our isolated finding suggests that SRS can be an effective treatment for macroprolactinomas that are only partially responsive to high-dosage dopamine-agonist therapy, and which enlarge on therapy. Careful initial evaluation (distance of the tumor margin from optic apparatus) is mandatory in order to assess the suitability of the technique [8-10]. Literature data show that the efficacy of SRS in the management of prolactinomas is variable and low [8-10]; this finding might possibly be related to the use of dopamine-agonists in these tumors [8-10]. The occurrence of thrombocytopenia in our patient did not seem to be related to SRS. Eight years after the diagnosis of partial HYPO with secondary hypothyroidism, the patient developed moderate hyperthyroidism, which was confirmed after L-thyroxine withdrawal. Thyroid USG did not show significant variations in nodule volume, and the last thyroid scan was comparable to the initial one (left autonomous nodule). Antithyroid drug therapy was required. A possible explanation for our patient's thyroid hormone modification over the years is that a pre-toxic adenoma with low secretory activity, initially masked by the coexistence of secondary hypothyroidism, increased its secretory activity over time, leading to hyperthyroidism.

## Conclusions

Our case highlights the importance of assessing free thyroid hormones whenever TSH levels are reduced. If both are reduced, suggesting secondary hypothyroidism, a thorough investigation of pituitary function for possible HYPO is warranted. Moderately increased PRL levels associated to voluminous pituitary tumors require serum dilution to avoid a possible hook effect and obtain a real (i.e. much higher) hormone value, which is indicative of macroprolactinoma. Macroprolactinomas are usually satisfactorily managed in terms of both tumor volume and PRL level reduction by long-acting dopamine agonist therapy. SRS may be a useful tool in those patients with macroprolactinoma who are resistant to medical therapy or when neurosurgery fails or the tumor is not amenable to neurosurgery. Hyperthyroidism secondary to toxic thyroid adenoma may coexist with hypopituitarism.

## Abbreviations

FSH: follicle stimulating hormone
GH: growth hormone
HYPO: hypopituitarism
IGF-I: Insulin-like growth factor 1
LH: luteinizing hormone
MRI: magnetic resonance imaging
PRL: prolactin
SRS: stereotactic radiosurgery
TSH: Thyroid stimulating hormone
USG: ultrasonography
